# Pathogenicity of Highly Pathogenic Avian Influenza Virus (H5N1) in Adult Mute Swans

**DOI:** 10.3201/eid1408.080078

**Published:** 2008-08

**Authors:** Donata Kalthoff, Angele Breithaupt, Jens P. Teifke, Anja Globig, Timm Harder, Thomas C. Mettenleiter, Martin Beer

**Affiliations:** * Friedrich-Loeffler-Institut, Greifswald-Insel Riems, Germany

**Keywords:** Mute swan, avian influenza, subtype H5N1, epidemiology, pathogenicity, preexposure immunity, dispatch

## Abstract

Adult, healthy mute swans were experimentally infected with highly pathogenic avian influenza virus A/*Cygnus cygnus*/Germany/R65/2006 subtype H5N1. Immunologically naive birds died, whereas animals with preexisting, naturally acquired avian influenza virus–specific antibodies became infected asymptomatically and shed virus. Adult mute swans are highly susceptible, excrete virus, and can be clinically protected by preexposure immunity.

Since 2002, highly pathogenic avian influenza (HPAI) subtype H5N1 viruses have spread from endemically infected areas of Southeast Asia to Europe and Africa and infected poultry and wild birds. Especially in Europe, swans proved to be the most frequently affected wild bird species (*1*). Recently, Brown et al. (*2*) inoculated juvenile mute swans and confirmed that they are the most likely swan species to transmit HPAI (H5N1). Nevertheless, it remains unclear if an age-related susceptibility exists as it does for ducks (*3*). In addition, a more detailed knowledge regarding the role of preinfection with low-pathogenicity avian influenza virus is required. Our study was designed to answer these questions by experimental infection of adult mute swans (*Cygnus olor*).

## The Study

All experiments with HPAI virus A/*Cygnus cygnus*/Germany/R65/2006 subtype H5N1 (*4*) were conducted under Biosaftey Level (BSL) 3+ conditions (trial approval LVL M-V/TSD/7221.3-1.1-003/07). The immunologically naive mute swans, 1–4 years of age, were divided into 2 groups. The high-dose group was inoculated oculo-oronasally with 10^6^ 50% egg infectious dose (EID)_50_/animal (n = 4); 1 additional naive contact swan was included in this group. The low-dose group received 10^4^ EID_50_/animal (n = 5); 2 additional swans were in contact with this group. Two additional animals had preexposure avian influenza virus–specific antibody titers and were included in the high-dose group; 1 was also dedicated as contact animal.

**Figure 1:**
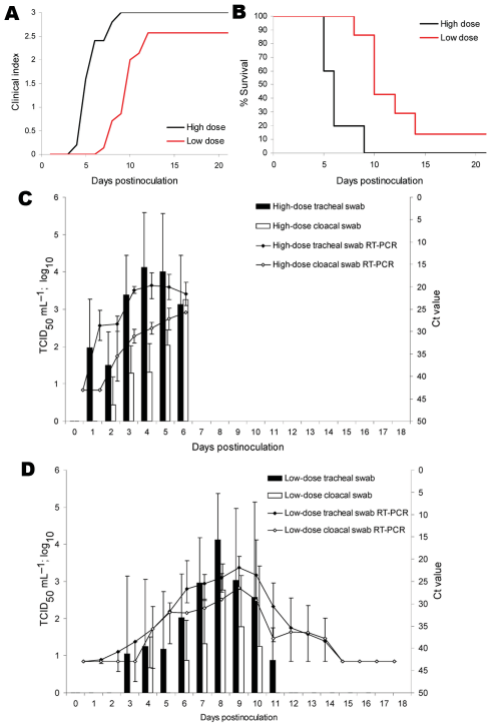
Clinical indices, mortality, and viral shedding of naive mute swans after inoculation with A/*Cygnus cygnus*/Germany/R65/2006 highly pathogenic influenza virus subtype H5N1. A) All animals were observed daily for up to 21 days for clinical signs and classified as healthy (0), ill (1), severely ill (2), or dead (3). A clinical index was calculated that represents the mean value of all naive swans per group for this period. B) Percentage survival of swans expressed as mean value of all naive swans per group. C and D) Mean values of the shedding of infectious virus of both groups (high dose = 10^6^ 50% egg infectious dose [EID_50_]/animal, and low dose = 10^4^ EID_50_/animal) of naive mute swans are shown. Mean cycle threshold (Ct) values of real-time reverse transcription–PCR (RT-PCR) analyses of tracheal and cloacal swabs are depicted for both groups. Standard deviations are shown as error bars. TCID_50_, 50% tissue culture infectious dose.

In most birds the clinical signs were inconspicuous after HPAI virus (H5N1) inoculation. However, 3 swans exhibited severe neurologic disorders, including opisthotonus, torticollis, and ataxia. In addition, 3 animals died suddenly without any clinical signs developing. The incubation period for the high-dose group was at least 4 days. All swans of this group died or had to be humanely killed 5–9 days postinoculation (DPI) (Figure 1, panels **A**, **B**). The minimum incubation period of the low-dose group was 7 days (Figure 1, panel **A**). Only 1 animal of the low-dose group survived until the end of the trial (21 DPI), and all other swans of the group succumbed between 8 and 14 DPI.

Oropharyngeal and cloacal swab samples were collected daily in Dulbecco modified Eagle medium supplemented with 5% fetal calf serum and antimicrobial drugs. All individual swabs were tested by real-time reverse transcription–PCR (*5*) specific for subtype H5N1, and the genomic load was semiquantified by the cycle threshold (Ct) value. Infectivity titers of swab samples were calculated as the 50% tissue culture infectious dose/mL on Madin-Darby canine kidney (MDCK) cells (collection of cell lines in veterinary medicine, Friedrich-Loeffler-Institut, Südufer Insel Riems, RIE83).

Viral RNA as well as replicating virus could be detected from 1 until 6 DPI in oropharyngeal swabs of the high-dose group (Figure 1, panel **C**). The Ct values ranged from 17 to 33. The swans of the low-dose group excreted infectious virus from 3 until 11 DPI in oropharyngeal swabs (Figure 1, panel **D**), and real-time reverse transcription–PCR detected viral RNA in this group from 3 until 14 DPI. Virus excretion from cloacal swabs was demonstrated from 2 to 6 DPI in the high-dose group (Figure 1, panel **C**) and 4–10 DPI in the low-dose group (Figure 1, panel **D**). Viral RNA detection from cloacal swabs were positive 3 days longer than titration in cell culture (low-dose group; Figure 1, panel **D**). Maximum duration of viral shedding per individual swan was 6 days in both groups for cloacal and tracheal swab samples. Virus excretion of the contact animals was delayed, but the quantities of excreted virus were similar to those of the inoculated animals. One adult male swan of the low-dose group was the only surviving naive swan; his excretion pattern was delayed and shortened compared to other swans, which received the same virus dosage.

Sera were collected at 0, 7, 14, and 21 DPI from surviving swans as well as on the day of euthanasia. Serum samples were heat inactivated at 56°C for 30 min and subsequently nucleoprotein antibody ELISA, serum neutralization test (SNT), and hemagglutination-inhibition (HI) test were performed. Results of serologic tests from the day of euthanasia or the last day of serum collection before death are shown in Table 1. The comparison of the 3 serologic tests showed that the nucleoprotein-specific ELISA was the most sensitive assay, showing positive results as early as 5 DPI. All but 4 swan sera were positive in the nucleoprotein antibody ELISA after HPAI virus (H5N1) inoculation. Six of 8 ELISA-positive serum samples also exhibited neutralizing antibodies, whereas only 4 of 8 ELISA-positive serum samples exhibited positive HI titers against the challenge virus antigen. However, even postinfection antibody titers >400 SNT or 128 HI, respectively, did not protect swans from dying (Table 1). The 1 surviving swan developed antibody titers >1,000 SNT or 500 HI at the end of the experiment.

**Table 1:** Distribution of viral genomic load and influenza A antigen in tissues of naive mute swans after challenge infection with highly pathogenic avian influenza virus (H5N1), related to assumed tropism and serologic data*

Test	Viral RNA load in tissue, Ct value†												
	High-dose group						Low-dose group						
	2	3	4‡	5	6		8‡	9‡	10	11	12§	13	14
Nasal concha	++	**+**	+	**++**	+		**+++**	/	**+++**	++	/	(+)	/
Trachea	/	**+**	/	(+)	(+)		(+)	/	**++**	(+)	/	(+)	/
Lung	(+)	**++**	++	+	(+)		**++**	/	**+++**	(+)	/	+	/
Brain	**++**	**++**	**(+)**	**+++**	**+**		**++**	**+**	**++**	**++**	/	**(+)**	**+**
Pancreas	**++**	**++**	+	**++**	**(+)**		**+++**	/	**++**	**+**	/	++	/
Adrenal gland	**++**	**+++**	(+)	**++**	(+)		**+++**	(+)	**+++**	(+)	/	+	/
Myocardium	/	**+**	(+)	+	/		+	/	**++**	(+)	/	/	(+)
Liver	+	**+++**	(+)	**+**	(+)		**+++**	/	**+++**	(+)	/	+	/
Kidney	(+)	**++**	(+)	(+)	/		++	(+)	**++**	(+)	/	+	(+)
Spleen	(+)	**+++**	(+)	+	(+)		++	(+)	**+++**	(+)	/	(+)	/
Bursa fabricii	/	**+**	+	(+)	+		**++**	/	++	+	/	+	(+)
Ovary/testis	**++**	**++**	(+)	**+++**	(+)		**+++**	/	**+++**	/	/	/	/
Proventriculus	(+)	**++**	(+)	(+)	(+)		++	(+)	**++**	+	/	(+)	+
Cecal tonsil	(+)	**++**	(+)	+	(+)		++	/	**++**	+	(+)	(+)	(+)
Tropism¶	N, EP	N, EP, EN	N	N, EP	N, EP		N, EP, EN	N, EP	N, EP, EN	N, EP	/	N	N
Serologic data													
ELISA	Pos	Neg	Pos	Pos	Neg		Neg	Pos	Neg	Pos	Pos	Pos	Pos
SNT	1	ND	1	5.3	ND		ND	5.7	ND	5.3	10.3	6.7	8.7
HI	2	ND	2	2	ND		ND	7	ND	2	9	8	7
DPI serology	5	0	7	6	0		7	12	7	7	21	10	14
Died or euthanized, DPI	5	5	9	6	6		10	12	10	8	21	10	14

*Viral RNA detected by real-time reverse transcription–PCR (RT-PCR) in swans after challenge infection with highly pathogenic avian influenza virus strain A/*Cygnus cygnus*/Germany/R65/06 (H5N1). N, neurotropism; EP, epitheliotropism; EN, endotheliotropism; ELISA, Pourquier AI A Blocking ELISA against nucleoprotein; Pos, positive; Neg, negative; SNT, serum neutralization test; [ND100 log2] modified from a previously described procedure (*6*); ND, not done; HI, hemagglutination-inhibition [log2] using homologous influenza virus (H5N1) as antigen according to standardized methods (*7*); DPI, days postinoculation. †Real-time RT-PCR results are presented as cycle of threshold (Ct) values: /, >40; (+), >30–40; +, >25–<30; ++, >20–<25; +++, <20. **Boldface** indicates marked positive staining by immunohistochemical analysis. ‡Contact animals. §Animal survived until the end of the study. ¶Tropism as assessed by immunohistochemical analysis.

Two mute swans showed positive or questionable results in a nucleoprotein-specific ELISA before inoculation. The neutralizing activity of sera of both animals against HPAI virus (H5N1) was low, with titers of 10 and 3, respectively; no specific HI titers were detected. After HPAI virus (H5N1) inoculation, both swans survived without any clinical signs. When these swans were compared with the inoculated naïve swans, viral shedding was delayed and had a shorter duration with reduced viral loads (Table 2).

**Table 2:** Viral shedding among mute swans with preexisting antibodies*

Individual serologically positive	Viral excretion, no. days shedding replication competent virus (DPI)			Genome detection, no. days with positive PCR results (DPI)			Peak titer log_10_/mL swab (DPI)			Minimum Ct value (DPI)	
OS	CS	OS	CS	OS	CS	OS	CS
No. 1 (contact)	1 (3)	0		7 (3-9)	6 (3-8)		1.75 (3)	0		32.12 (4)	31.65 (4)
No. 7	4 (2–5)	1 (4)		8 (1–8)	6 (3–7, 9)		2.63 (4)	1.75 (4)		26.44 (5)	29.49 (4)

*H5-specific real-time reverse transcription–PCR (RT-PCR) results of swab samples from swans. DPI, days postinoculation; Ct value, cycle threshold of the real-time RT-PCR; OS, oropharyngeal swab; CS, cloacal swab.

**Figure 2:**
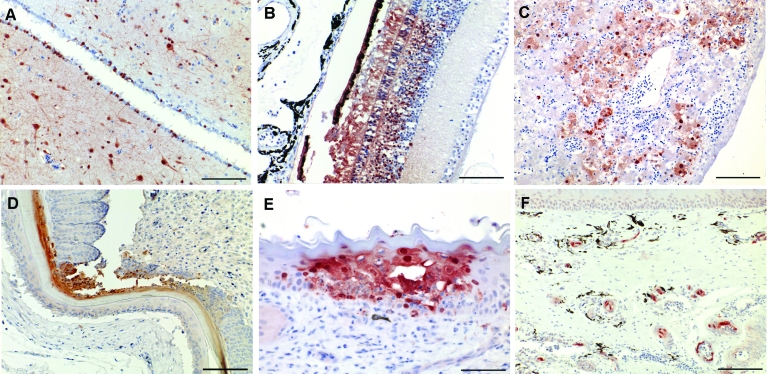
Immunohistochemical analysis for nucleoprotein of avian influenza virus. Tissue sections were stained by using the avidin-biotin-peroxidase complex method, 3-amino-9-ethylcarbazole (red), and hematoxylin (blue). A) Brain, cerebrum: numerous glial cells, neurons and ependymal cells stain positive for influenza virus antigen (scale bar = 200 μm). B) Eye, retina: cells of the pigmented epithelial layer, photoreceptor cells, and cells of the outer and inner nuclear layers are positive for the nucleoprotein of influenza virus (scale bar = 100 μm). C) Liver: subadjacent to the capsule there is hepatocyte degeneration and necrosis around a congested central vein (scale bar = 100 μm). D) Skin: keratinized layer of the feather follicular epithelium shows focal necrosis with intense nuclear and cytoplasmic immunostaining (scale bar =100 μm). E) Nasal cavity: focal intraepithelial necrosis of the mucocutaneous membrane associated with influenza virus infection (scale bar = 50 μm). F) Nasal concha: numerous submucosal arterioles and venules display strong endothelial staining, which partially extends into the media of the vessels (scale bar = 100 μm).

Gross pathology showed widespread hemorrhages as predominant lesions in both infected groups. Ecchymoses were especially present within the myocardium, submeningeally in the brain, in the peritracheal connective tissue, and within the lungs. Petechiae were seen in the pancreas, liver, and subcutis and on serosal surfaces. Only 2 swans exhibited multifocal to coalescent foci of coagulative necrosis in the pancreas. Table 1 summarizes semiquantified viral RNA loads in comparison to immunohistochemical detection of avian influenza virus nucleoprotein in different tissues. Immunostaining for avian influenza virus antigen was positive in 11 of 14 animals and confined to 3 locations: neuronal, epithelial, and endothelial (Figure 2, Table 1). In all animals, a strong neuronal infection could be observed with viral antigen in the cytoplasm and nuclei of neurons, glial cells, and ependymal cells in the brain, spinal cord, and eye (Table 1, Figure 2, panels **A**, **B**). Peripheral nerves, e.g., innervating the adrenal glands, the ovary, or area located adjacent to the cecal tonsil also stained positive. Some swans showed immunoreactivity in epithelial cells, e.g., of the pancreas, adrenal glands, ovaries, liver (Figure 2, panel **C**), feather follicles, and nasal cavity (Figure 2, panels **D**, **E**). Endothelia of different organs stained strongly positive only in 3 swans (Figure 2, panel **F**); in addition, these animals exhibited very high loads of viral genome in all tissue samples (Table 1).

## Conclusions

We demonstrated that few adult mute swans might have the ability to survive infection with HPAI virus (H5N1). Surviviors would most likely be older swans in good health infected with a low dosage (e.g., <10^4^ EID_50_/animal). However, because of viral shedding for several days without showing severe clinical symptoms, adult mute swans could play a key role in the spread of HPAI virus (H5N1), a conclusion that contradicts those of other investigators (*8*–*10*). Gross and histologic lesions in infected swans were independent of dosage, age, or sex of infected swans. Two parallel courses of pathogenesis with predominantly endothelial (n = 3) or epithelial/neuro­nal (n = 9) infections were distinguishable. Both forms have been described (*11*). However, normally only 1 dominant type was observed, depending on the experimental conditions (e.g., species, age of the animals, virus strain). At least 2 swans with the endotheliotropic course of infection were negative for avian influenza virus–specific antibodies. Thus, failure to mount an early antibody response might be responsible for or promote infection of the vasculature. In contrast, preexisting avian influenza virus–specific antibodies can be an efficient modulator of the outcome of an infection with HPAI virus (H5N1).
